# Human Genetic Disorders and Knockout Mice Deficient in Glycosaminoglycan

**DOI:** 10.1155/2014/495764

**Published:** 2014-07-13

**Authors:** Shuji Mizumoto, Shuhei Yamada, Kazuyuki Sugahara

**Affiliations:** ^1^Department of Pathobiochemistry, Faculty of Pharmacy, Meijo University, 150 Yagotoyama, Tempaku-ku, Nagoya 468-8503, Japan; ^2^Laboratory of Proteoglycan Signaling and Therapeutics, Frontier Research Center for Post-Genomic Science and Technology, Graduate School of Life Science, Hokkaido University, West-11, North-21, Kita-ku, Sapporo, Hokkaido 001-0021, Japan

## Abstract

Glycosaminoglycans (GAGs) are constructed through the stepwise addition of respective monosaccharides by various glycosyltransferases and maturated by epimerases and sulfotransferases. The structural diversity of GAG polysaccharides, including their sulfation patterns and sequential arrangements, is essential for a wide range of biological activities such as cell signaling, cell proliferation, tissue morphogenesis, and interactions with various growth factors. Studies using knockout mice of enzymes responsible for the biosynthesis of the GAG side chains of proteoglycans have revealed their physiological functions. Furthermore, mutations in the human genes encoding glycosyltransferases, sulfotransferases, and related enzymes responsible for the biosynthesis of GAGs cause a number of genetic disorders including chondrodysplasia, spondyloepiphyseal dysplasia, and Ehlers-Danlos syndromes. This review focused on the increasing number of glycobiological studies on knockout mice and genetic diseases caused by disturbances in the biosynthetic enzymes for GAGs.

## 1. Introduction

**Figure 1:**
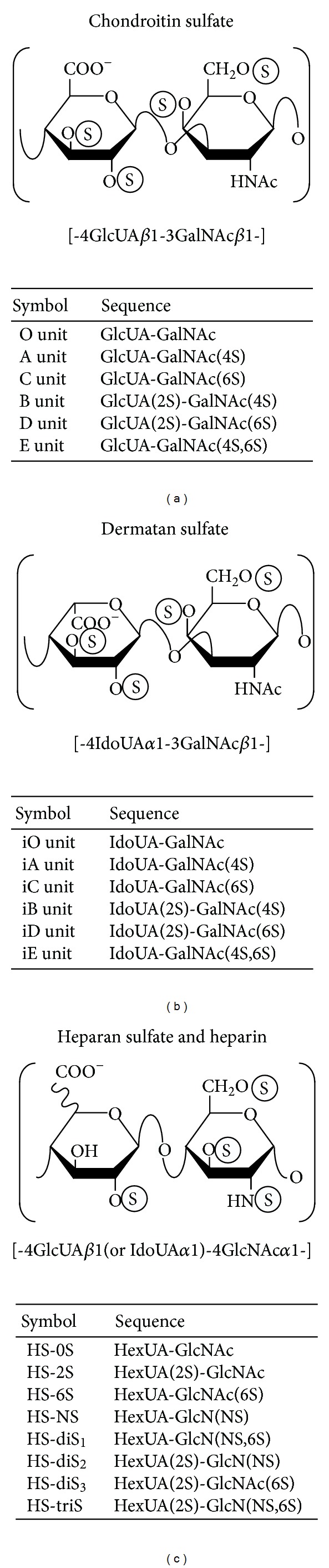
Typical repeating disaccharide units in CS, DS, HS, and heparin, and their potential sulfation sites. CS consists of GlcUA and GalNAc, whereas DS is a stereoisomer of CS including IdoUA instead of GlcUA. Both linear polysaccharides are often found as CS-DS hybrid chains in mammals. HS and heparin consist of uronic acid and GlcNAc residues with varying proportions of IdoUA. Heparin is highly sulfated and has a large proportion of IdoUA residues, whereas HS is low sulfated and has a high proportion of GlcUA. These sugar moieties are esterified by sulfate at various positions as indicated by the circled “S.” The abbreviation of “i” in iO, iA, iB, iC, iD, and iE stands for IdoUA. HexUA represents hexuronic acid (GlcUA or IdoUA).

Glycosaminoglycans (GAGs) are covalently attached to the core proteins that form proteoglycans (PGs), which are ubiquitously distributed in extracellular matrix and on the cell surface [1–7]. GAGs are linear polysaccharides that form the side chains of PGs and have been classified into chondroitin sulfate (CS), dermatan sulfate (DS), heparan sulfate (HS), and heparin based on their structural units. The backbone of CS consists of repeating disaccharide units of* N*-acetyl-d-galactosamine (GalNAc) and d-glucuronic acid (GlcUA) (Figure 1). DS is a stereoisomer of CS and composed of GalNAc and l-iduronic acid (IdoUA) instead of GlcUA (Figure 1). They are often distributed as CS-DS hybrid chains in mammalian tissues [8]. On the other hand, HS and heparin consist of* N*-acetyl-d-glucosamine (GlcNAc) and GlcUA or IdoUA (Figure 1). The glucosamine (GlcN) residues in HS and heparin are modified by not only* N*-acetylation but also* N*-sulfation. These GAG chains are modified by sulfation at various hydroxy group positions and also by the epimerization of uronic acid residues during the biosynthetic process, thereby giving rise to structural diversity, which plays an important role in a wide range of biological roles including cell proliferation, tissue morphogenesis, infections by viruses, and interactions with various growth factors, cytokines, and morphogens [7–18].

**Figure 2:**
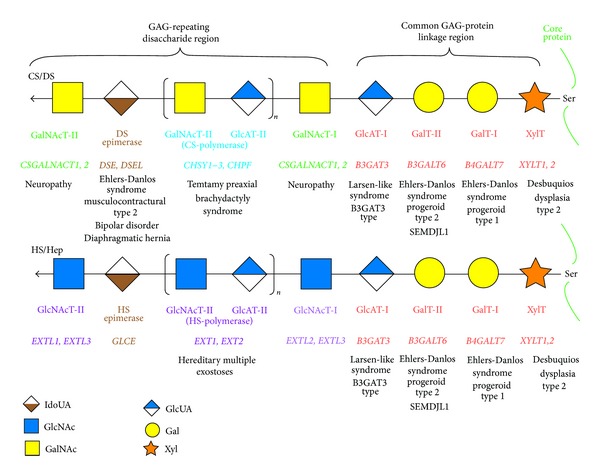
Biosynthetic assembly of GAG backbones by various glycosyltransferases. All glycosyltransferases require a corresponding UDP-sugar, such as UDP-Xyl, -Gal, -GlcUA, -GalNAc, and -GlcNAc, as a donor substrate. After specific core proteins have been synthesized, the synthesis of the common GAG-protein linkage region, GlcUA*β*1-3Gal*β*1-3Gal*β*1-4Xyl*β*1-, is evoked by XylT, which transfers a Xyl residue from UDP-Xyl to the specific serine (Ser) residue(s) at the GAG attachment sites. The linkage tetrasaccharide is subsequently constructed by GalT-I, GalT-II, and GlcAT-I. These four enzymes are common to the biosynthesis of CS, DS, HS, and heparin. The first *β*1-4-linked GalNAc residue is then transferred to the GlcUA residue in the linkage region by GalNAcT-I, which initiates the assembly of the chondroitin backbone, thereby resulting in the formation of the repeating disaccharide region, [-3GalNAc*β*1-4GlcUA*β*1-]_*n*_, by CS-polymerase. Alternatively, the addition of *α*1-4-linked GlcNAc to the linkage region by GlcNAcT-I initiates the assembly of the repeating disaccharide region [-4GlcNAc*α*1-4GlcUA*β*1-]_*n*_ of HS and heparin by HS-polymerase. Following the formation of the chondroitin and heparan backbones, both precursor chains are modified by sulfation and epimerization (see Figure 3). Each enzyme, its coding gene, and the corresponding inheritable disorder are described under the respective sugar symbols from the top of each line. SEMDJL1, spondyloepimetaphyseal dysplasia with joint laxity type 1.

**Figure 3:**
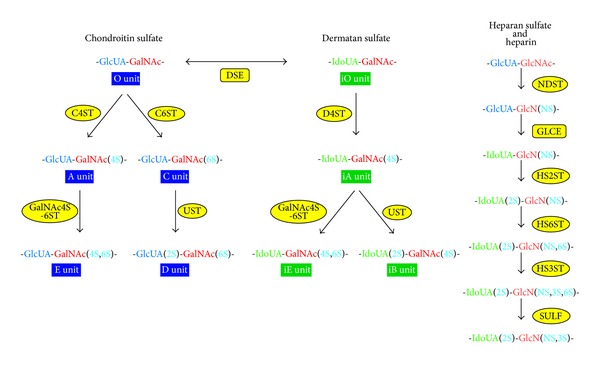
Modification pathways of CS, DS, HS, and heparin. After formation of the GAG backbones, including chondroitin and heparan, each sugar residue is modified by sulfation, which is catalyzed at various positions by sulfotransferases, as indicated in the figure. C4ST and C6ST transfer a sulfate group from PAPS to the C-4 or C-6 position of the GalNAc residues in the CS chain, which results in the formation of A-units and C-units, respectively. Further sulfations are catalyzed by GalNAc4S-6ST or UST, which is required for the formation of disulfated disaccharide units, E-units and D-units, respectively. DS-epimerase converts GlcUA into IdoUA by epimerizing the C-5 carboxy group in the chondroitin precursor, thereby resulting in the formation of the dermatan backbone. D4ST, which is distinct from C4ST, transfers a sulfate group from PAPS to the C-4 position of the GalNAc residues in dermatan to form the iA-units. The disulfated disaccharide units, iB and iE, are infrequently synthesized by UST and GalNAc4S-6ST, which are the same enzymes as those responsible for the biosynthesis of B and E units in CS chains. Following the synthesis of the backbone of HS or heparin by HS polymerases, the first modifications,* N*-deacetylation and* N*-sulfation, are catalyzed by NDST. Some GlcUA residues are then converted to IdoUA residues by GLCE. Thereafter, the hydroxy groups at the C-2 of IdoUA and at C-3 and C-6 of* N*-sulfated glucosamine and/or GlcNAc are sulfated by specific sulfotransferases. The 6-*O*-desulfation of the* N*-sulfated GlcN residue in the HS and heparin chains occurs by the action of SULF in order to modify the fine structure of HS for the regulation of interactions with various signaling molecules. C4ST, chondroitin 4-*O*-sulfotransferase; C6ST, chondroitin 6-*O*-sulfotransferase; D4ST, dermatan 4-*O*-sulfotransferase; DSE, dermatan sulfate C5-epimerase; GalNAc4S-6ST, GalNAc 4-sulfate 6-*O*-sulfotransferase; GLCE, heparan sulfate C5-epimerase; HS2ST, heparan sulfate 2-*O*-sulfotransferase; HS3ST, heparan sulfate 3-*O*-sulfotransferase; HS6ST, heparan sulfate 6-*O*-sulfotransferase; NDST,* N*-deacetylase/*N*-sulfotransferase; UST, uronyl 2-*O*-sulfotransferase;* N*S, 2S, 4S, and 6S, 2-*N*-, 2-*O*-, 4-*O*-, and 6-*O*-sulfate, respectively.

Glycosyltransferases, epimerases, sulfotransferases, and related enzymes in the biosynthesis of GAGs have been cloned and characterized (Tables 1–4 and Figures 2 and 3) [6, 7, 14, 19]. Furthermore, genetic analyses using model animals including mice, zebrafish, fruit flies, and nematodes have led to new findings on different phenotypes [4, 8, 9, 12, 13]. Human genetic disorders including bone and skin diseases caused by mutations in the genes encoding the biosynthetic enzymes for GAGs have recently been reported [7, 14, 20]. This review focused on recent advances in knockout mice for GAG biosynthesis, as well as cartilage and connective tissue disorders caused by disturbances in the biosynthesis of functional GAG chains.

## 2. Biosynthesis of 3′-Phosphoadenosine 5′-Phosphosulfate

The sulfation of GAGs is required for the exertion of their physiological functions. Sulfotransferases catalyze the transfer of sulfate from the donor substrate, 3′-phosphoadenosine 5′-phosphosulfate (PAPS), to the corresponding acceptor substrates [21]. PAPS is synthesized from ATP and inorganic sulfate in the cytosol, and the reaction takes place in two sequential steps [21–23]. ATP sulfurylase firstly catalyzes the reaction between ATP and inorganic sulfate to form the biosynthetic intermediate, adenosine 5′-phosphosulfate (APS) [22, 23]. The formation of the active sulfate, PAPS, is then catalyzed by APS kinase, which involves a reaction between APS and ATP [22, 23]. ATP sulfurylase and APS kinase are encoded by the respective genes in bacteria, fungi, yeast, and plants [21]. On the other hand, both enzymes are fused in animals, resulting in a polypeptide designated PAPS synthase (PAPSS), which is a bifunctional enzyme composed of the N-terminal APS kinase domain and C-terminal ATP sulfurylase domain [21]. Following the formation of PAPS in the cytosol, PAPS is translocated into the Golgi by PAPS transporters [24].

## 3. Biosynthesis of GAG Chains

### 3.1. GAG-Protein Linkage Region

CS, DS, HS, and heparin chains are attached to serine residues in core proteins through the common GAG-protein linkage region tetrasaccharide, GlcUA*β*1-3galactose*β*1-3galactose*β*1-4xylose*β*1-*O*- (GlcUA-Gal-Gal-Xyl-*O*-) (Figure 2) [1, 5]. The transfer of a Xyl residue from uridine diphosphate (UDP)-Xyl to specific serine residues in the newly synthesized core proteins of PGs in the endoplasmic reticulum and* cis*-Golgi compartments is initiated by *β*-xylosyltransferase (XylT) (Figure 2 and Table 2) [25, 26]. *β*1,4-Galactosyltransferase-I (GalT-I), which is encoded by* B4GALT7*, then transfers a Gal residue from UDP-Gal to the Xyl-*O*-serine in the core proteins [27, 28]. *β*1,3-Galactosyltransferase-II (GalT-II), which is encoded by* B3GALT6*, transfers another Gal residue from UDP-Gal to the Gal-Xyl-*O*-serine [29]. Finally, *β*1,3-glucuronosyltransferase-I (GlcAT-I), which is encoded by* B3GAT3*, transfers a GlcUA residue from UDP-GlcUA to the Gal-Gal-Xyl-*O*-serine (Figure 2 and Table 2) [30]. These enzymes may form a multienzyme complex such as the so-called GAGosome for GAG synthesizing enzymes for the construction of the linkage region [31, 32].

Several modifications including the 2-*O*-phosphorylation of the Xyl residue as well as sulfation at the C-6 position of the first Gal and at C-4 or C-6 of the second Gal residue have been reported [5]. GAG-Xyl kinase, encoded by* FAM20B*, Xyl phosphatase, encoded by* ACPL2*, and Gal-6-*O*-sulfotransferase, encoded by* CHST3 (C6ST1),* have so far been identified (Table 2) [33–35]. These modifications affect the glycosyltransferase reactions of GalT-I and GlcAT-I* in vitro* and may regulate the formation of GAG chains [36, 37].

### 3.2. Repeating Disaccharide Region of CS and DS

Chain polymerization of the repeating disaccharide region in CS and DS chains is initiated by the transfer of the first GalNAc from UDP-GalNAc to the GlcUA residue in the linkage region tetrasaccharide, GlcUA-Gal-Gal-Xyl-*O*-, by *β*1,4-*N*-acetylgalactosaminyltransferase-I (GalNAcT-I) (Figure 2) [193–196]. Alternatively, the transfer of a GlcNAc residue from UDP-GlcNAc to the linkage region tetrasaccharide by *α*1,4-*N*-acetylglucosaminyltransferase-I (GlcNAcT-I) is known to result in the initiation of the repeating disaccharide region of HS and heparin chains (Figure 2) [197–201]. Six chondroitin synthase family members have been identified including chondroitin synthases (ChSys), chondroitin-polymerizing factor (ChPF), and CSGalNAcTs (Figure 2 and Table 3) [193–196, 202–208]. ChSy1 is composed of 802 amino acids and is a bifunctional glycosyltransferase that exhibits CS-GlcAT-II and GalNAcT-II activities, which are required for the biosynthesis of the repeating disaccharide region, -4GlcUA*β*1-3GalNAc*β*1 (Table 3) [202]. ChSy1 itself is unable to construct the backbone of CS by the activity of polymerase, whereas the enzyme complex of ChSy with ChPF can form the repeating disaccharide region [203–205]. A precursor of CS, the chondroitin backbone, is then maturated by sulfation modified by various sulfotransferases such as uronosyl 2-*O*-sulfotransferase (UST) [209], chondroitin 4-*O*-sulfotransferases (C4ST) [210–212], chondroitin 6-*O*-sulfotransferase (C6ST) [213, 214], and GalNAc 4-sulfate 6-*O*-sulfotransferase (GalNAc4S-6ST) [215] (Figure 3 and Table 3). These transfer the sulfate group from the sulfate donor PAPS to the corresponding position of the GlcUA and GalNAc residues in chondroitin. C4STs have been shown to regulate the chain length and amount of CS coordinating with CSGalNAcTs [216, 217].

Epimerization of the C-5 position of GlcUA residues in a chondroitin polymer as a precursor backbone occurs during or after the chain elongation, which results in the formation of the repeating disaccharide region, -4IdoUA*α*1-3GalNAc*β*1-, of DS chains (Figure 3) [218–221]. The dermatan chains fully develop through sulfation catalyzed by dermatan 4-*O*-sulfotransferases (D4ST) [222, 223] and uronosyl 2-*O*-sulfotransferase (UST) [209] (Figure 3 and Table 3).

### 3.3. Repeating Disaccharide Region of HS and Heparin

Following the construction of the linkage region tetrasaccharide, GlcUA*β*1-3Gal*β*1-3Gal*β*1-4Xyl*β*1-*O*-serine, on the core protein, transfer of the GlcNAc residue from UDP-GlcNAc to the tetrasaccharide induces chain polymerization of the repeating disaccharide region of HS and heparin catalyzed by GlcNAcT-I [197–201] (Figure 2). After the addition of the first GlcNAc to the linkage region, the growing pentasaccharide is further elongated by alternate additions of GlcUA and GlcNAc from UDP-GlcUA and UDP-GlcNAc by HS-*β*1,4glucuronyltransferase-II (HS-GlcAT-II) and *α*1,4-*N*-acetylglucosaminyltransferase-II (GlcNAcT-II), respectively (Figure 2). Exostosin 1 (EXT1) as well as 2 (EXT2) both exhibit HS-GlcAT-II and GlcNAcT-II activities [199, 224–226] (Table 4). Furthermore, the heterodimeric complex of EXT1 and EXT2 exhibits HS polymerase activity on a linkage region tetrasaccharide acceptor* in vitro*, which results in the biosynthesis of HS and heparin polysaccharides [227, 228]. Three homologous genes to the* EXT* have been identified [6, 14, 229]. EXTL1 and EXTL2 exhibit GlcNAcT-II and GlcNAcT-I activities, respectively, whereas EXTL3 has not only GlcNAcT-I, but also GlcNAcT-II activities (Figure 2 and Table 4) [200, 201].

After the formation of the repeating disaccharide backbone of HS chains by EXTs and EXTLs, GlcNAc residues are converted into GlcN residues by GlcNAc* N*-deacetylase (Figure 3) [6, 14, 198]. A sulfate group is subsequently transferred from PAPS to the GlcN by GlcN* N*-sulfotransferase [6, 14, 198]. Both enzymes are encoded by a single gene,* GlcNAc N-deacetylase/N-sulfotransferase* (Figure 3 and Table 4) [230–233]. The interconversion of GlcUA to IdoUA in HS and heparin is achieved by HS-glucuronyl C5-epimerase (Figure 3) [234–236]. Moreover, sulfation at the C-2 position of uronic acid as well as C-3 and C-6 positions of the GlcN residues in the HS and heparin are catalyzed by HS 2-*O*-sulfotransferase, HS 3-*O*-sulfotransferase, and HS 6-*O*-sulfotransferase, respectively (Figure 3 and Table 4) [237–244]. The desulfation of 6-*O*-sulfated GlcNS residues in HS chains by HS 6-*O*-endosulfatase modifies the fine structure of HS in order to regulate various biological events including cell signaling, tumor growth, and angiogenesis (Figure 3 and Table 4) [245–247].

## 4. Knockout and Transgenic Mice of GAG Biosynthetic Enzymes

### 4.1. Xylt1

A recessive dwarf mouse mutant (*pug*) obtained from an* N*-ethyl-*N*-nitrosourea mutagenesis screen was attributed to a missense mutation in* Xylt1*, which resulted in the substitution of an amino acid (p.Trp932Arg) [59]. XylT activity in the* pug* mutant was markedly reduced* in vitro*, which resulted in a decrease in the amount of GAGs in cartilage. Furthermore, early ossification was reported in this mutant, which resulted in a shorter body length than that of a wild-type embryo. These phenotypes may be caused by an upregulation of Indian hedgehog signaling but not MAPK signaling due to lack of GAGs [59].

### 4.2. *B3gat3* (GlcAT-I)

Mice deficient in* GlcAT-I* synthesize a smaller CS and HS chain in their blastocysts than that of the heterozygous mice [75]. In addition, these mice exhibit an embryonic lethality before the 8-cell stage due to the failure of cytokinesis, which has been attributed to a deficiency in CS, but not HS based on the findings reported in embryos treated with chondroitinase and heparinase [76]. Moreover, interaction of CS with E-cadherin, which regulates the differentiation of embryonic stem cells, may control Rho signaling pathway [76]. These findings indicated that CS, but not HS, is involved in regulating cell division in mammals.

### 4.3. Csgalnact1 and Csgalnact2

*CSGalNAcT1*-null mice have been shown to produce a smaller amount as well as a shorter length of CS chains than the wild-type [84, 85]. These mice also have shorter limbs and axial skeleton and a thinner growth plate in cartilage than wild-type mice, which results in a slightly shorter body length and smaller body weight [84, 85]. It is likely that the reduction in CS may affect normal chondrogenesis and formation of type II collagen fibers [84]. These findings suggest that CSGalNAcT1 is essential for the differentiation and maturation of cartilage.

A deficiency in CSGalNAcT1, but not CSGalNAcT2, has been shown to promote axonal regeneration following spinal cord injury [86]. CS-PGs function as barrier-forming molecules during axonal regeneration after damage to the nervous system [10]. Thus, the down- and upregulation of CS and HS biosynthesis, respectively, in the scars of *CSGalNAcT*1^−/−^ mice led to better recovery from injuries in the nervous system than the wild type.

### 4.4. Chsy1

*Chsy1*-deficient mice are viable but exhibit chondrodysplasia, progression of the bifurcation of digits, delayed endochondral ossification, and reduced bone density [81]. Furthermore, a decrease in 4-*O*-sulfation and increase in 6-*O*-sulfation as well as desulfation of the GalNAc residues of CS have been reported in the cartilage of* Chsy1* ^−/−^ mice. The signaling of hedgehog but not of FGF, bone morphogenetic protein, or transforming growth factor-*β* altered in primary chondrocytes from Chsy1-deficient mice [81], which suggests that CS-PGs and hedgehog protein may coordinately regulate skeletal development and digit patterning.

### 4.5. Chpf

Mice deficient in* Chpf*, also known as* chondroitin sulfate synthase-2* (*CSS2*), are fertile and viable and exhibit no obvious abnormalities including osteoarthritis and cartilage development [82]. These findings are consistent with the study by Wilson et al. [81].

### 4.6. Dse and Dsel

The body weight of* Dse* ^−/−^ mutant mice, which have fewer IdoUA residues in the skin, is ~30% smaller than that of the wild type [88, 89]. Although no significant differences were observed in the content of collagen between* Dse* ^−/−^ and the wild type, the ultrastructure of collagen fibrils in the dermis and hypodermis was thicker in the deficient mice than in the wild type, and a decline in their mechanical strength was also noted in the deficient mice. On the other hand, no morphological or histological abnormalities have been reported in mice targeted with the disruption of DS epimerase-2 encoded by* Dsel* [93]. In addition, 4-*O*-sulfation of the DS chain was decreased in the brain of* Dse2* ^−/−^, whereas the adult* Dse2* ^−/−^ brain had normal structures in the extracellular matrix. The function of Dse2 appears to be compensated by Dse1 [93].

### 4.7. Chst3 (C6st1)

The number of 6-*O*-sulfated disaccharide units including the C-unit (GlcUA–GalNAc6-*O*-sulfate) and D-unit (GlcUA2-*O*-sulfate–GalNAc6-*O*-sulfate) was shown to be markedly reduced in the spleens and brains of* C6st1*-deficient mice, and the number of naive T lymphocytes was also decreased in the spleen [114]. However, brain development in* C6st1* ^−/−^ mice is normal in spite of a decrease in D-units in the CS chains of the null mice.

CS-PGs are newly synthesized in the central nervous system following injury, and this inhibits axonal regeneration [10, 248]. Furthermore, upregulation of the expression of* C6st1* and 6-*O*-sulfated CS-PGs has been demonstrated in glial scars after a cortical injury [249].* C6st1* ^−/−^ mice had fewer or a similar number of regenerative axons after axotomy to the wild type [115].

An increase in chondroitin 6-*O*-sulfation was observed in the developing brains of* C6st1*-transgenic mice and affected the formation of the perineuronal nets and cortical plasticity [116], which are specialized structures of the dense organized matrix, which are composed of CS-PGs, hyaluronan, tenascins, and link proteins and regulate neuronal plasticity and neuroprotection [250]. Chondroitin 6-*O*-sulfate may regulate the maturation of parvalbumin-expressing interneurons through the incorporation of Otx2 [116], which regulates ocular dominance plasticity.

### 4.8. Chst11 (C4st1)

The* C4st1* gene was identified as a target gene of bone morphogenetic protein signaling using gene trap experiments [94].* C4st1*-mutant mice exhibit severe dwarfism and die within six hours of birth due to respiratory failure [95]. Moreover, severe chondrodysplasia with abnormalities in the cartilage growth plate and chondrocyte columns, marked reductions in GAG content and 4-*O*-sulfated CS, the downregulation of bone morphogenetic protein signaling, and the upregulation of transforming growth factor-*β* have been observed in these mice. These findings indicated that C4ST1 and the 4-*O*-sulfation of CS chains were essential for the signaling pathways of bone morphogenetic protein and transforming growth factor-*β* as well as cartilage morphogenesis.

### 4.9. *Chst14* (D4st1)

*D4st1* ^−/−^ mice have a smaller body weight, a kinked tail, and more fragile skin and are less fertile than the wild type. [107]. In addition, axonal regrowth is initially facilitated in* D4st1* ^−/−^ mice following nerve transection.

Furthermore, the impaired proliferation of neural stem cells, reduced neurogenesis, and an altered subpopulations of radial glial cells have been reported in* D4st1*-deficient mice [96]. The epitope structure recognized by the monoclonal anti-CS antibody 473HD, which contains the D-unit (GlcUA-2-*O*-sulfate–GalNAc-6-*O*-sulfate) and iA-unit (IdoUA–GalNAc4-*O*-sulfate) in the CS-DS hybrid chains on PGs, such as phosphacan, is required for the formation of neurospheres and as a marker for radial glial cells [251]. Expression of the 473HD epitope was shown to be decreased in the neural stem cells of* D4st1* ^−/−^ mice, and this resulted in the altered formation of neurospheres [96]. These findings indicated that DS chains and/or D4ST1 are essential for the proliferation and differentiation of neural stem cells.

### 4.10. Chst15 (Galnac4s-6st)

*Galnac4s-6st*-null mice are viable and fertile and completely defective in the E-unit, GlcUA-GalNAc(4-,6-*O*-disulfates), in both CS and DS chains [117]. The activities of carboxypeptidase A and tryptase from bone marrow-derived mast cells in* Galnac4s-6st* ^−/−^ were lower than those in the wild type, which suggested that the E-unit-containing CS chain or CS-PGs may be involved in the retention of these proteases in the granules of mast cells.

### 4.11. Ext1 and Ext2

Gene knockout mice produced by the targeted disruption of the gene encoding* Ext1* and* Ext2* died by embryonic day 8.5–14.5 due to defects in the formation of the mesoderm and a failure in egg cylinder elongation [119–121, 136]. The GlcUA and GlcNAc transferase activities are decreased and HS chains are shorter in mice carrying a hypomorphic mutation in* EXT1* generated by gene trapping, which affect the signaling pathways of Indian hedgehog and parathyroid hormone-related peptide [120, 121]. Thus, it is difficult to analyze the* in vivo* functions of HS chains using conventional knockout mice. A growing number of conditional knockout mice produced by targeted disruption of the gene encoding HS biosynthetic enzymes has provided an insight into the physiological functions of HS and HS-PGs [14]. For example, pluripotent embryonic stem cells in which* Ext1* was disrupted fail to differentiate into neural precursor cells and mesoderm cells due to the enhancement of Fgf signaling and retention of the high expression of* Nanog* [122, 123]. Conditional* Ext1*-knockout mice selectively disrupted in the nervous system die within the first day of life and have defective olfactory bulbs, midbrain-hindbrain region, and axon guidance due to a disturbance in signaling pathways including Fgf8 and Netrin-1 [124–126]. Conditional* Ext1*-knockout mice specific for postnatal neurons exhibit a large number of autism-like phenotypes in spite of a normal morphology in the brain [127]. On the other hand, mice in which* Ext1* was specifically disrupted for chondrocytes and the limb bud,* Ext2* heterozygous mice, and compound* Ext1* ^+/−^/*Ext2* ^+/−^ mice display severe skeletal defects with cartilage differentiation and chondrocyte maturation, and these defects resembled an autosomally dominant inherited genetic disorder, human hereditary multiple exostoses [128–132]. Disruption of the* Ext1* gene in glomerular podocytes results in an abnormal morphology in these cells [133]. Furthermore, conditional knockout mice lacking* Ext1* in the high endothelial venules and vascular endothelium cells show a decrease in lymphocyte homing to peripheral lymph nodes and a compromised contact hypersensitivity response [134, 135]. These findings suggest that HS and HS-PGs are essential for playing a role in their physiological functions in a tissue-specific manner.

### 4.12. Extl2 and Extl3

Mice deficient in* Extl2* are viable and develop normally; however, they produce a larger amount of GAG chains [137, 138]. Liver regeneration was shown to be impaired in these knockout mice following liver injury induced by administration of CCl_4_ due to suppression of the response to hepatocyte growth factor [137].

Mice deficient in* Extl3* are embryonically lethal, which is similar to mice lacking* Ext1* or* Ext2* [139]. In addition, selective inactivation of the* Extl3* gene in pancreatic islet *β*-cells caused an abnormal morphology as well as a reduction in the proliferation of the islets, which resulted in defective insulin secretion [139]. However, it remains to be determined how HS, HS-PGs, or Extl3 is involved in insulin secretion.

### 4.13. *Ndst1*,* 2*, and* 3*

Functional analyses of HS and heparin using* Ndst1*-deficient mice have been performed in approximately 20 studies to date [140–164]. Representative studies have been reviewed in this chapter.* Ndst1*-deficient mice die after birth and have cerebral hypoplasia, axon guidance errors, defects in the eye and olfactory bulbs, insufficient milk production caused by a defect in lobuloalveolar expansion in the mammary gland, and morphological abnormalities in the podocytes [140–142, 145, 156, 157, 162, 164].* Ndst1* conditional knockout mice specific for the liver accumulated triglyceride-rich lipoproteins due to a reduction in the clearance of cholesterol-rich lipoprotein particles [148, 163]. Furthermore, mice with the endothelial-targeted deletion of* Ndst1* exhibited suppressed experimental tumor growth and angiogenesis including microvascular density and branching of the surrounding tumors due to altered responses to Fgf2 and Vegf, which resulted in reduced Erk phosphorylation [147] and attenuated allergic airway inflammation [151].

Embryos from* Ndst2*-deficient mouse are viable and fertile, whereas their mast cells are unable to synthesize heparin, which leads to changes in morphology and severely reduced amounts of granule proteases [165–167]. These findings indicated that the storage of proteases in granules is controlled by heparin or heparin-PG, such as serglycin [165–167]. On the other hand,* Ndst3*-deficient mice develop normally and are fertile [168].

### 4.14. Glce (HS GlcUA C5-epimerase)

Mice with the targeted disruption of* HS epimerase* die immediately after birth and have agenesis of the kidney, a shorter body length, and lung defects [169, 170]. Furthermore, developmental abnormalities in the lymphoid organs, including the spleen, thymus, and lymph nodes, have been reported in the knockout mice [171, 172]. IdoUA-containing HS chains are critical for early morphogenesis of the thymus through binding with Fgf2, Fgf10, and bone morphogenetic protein 4 [171]. In addition, the interaction of HS with a proliferation inducing ligand, hepatocyte growth factor, and CXCL12*α* is required for B-cell maturation [172].

### 4.15. Hs2st

Gene trap mice lacking* Hs2st* die during the neonatal period and exhibit renal aplasia and defects in the eyes, skeleton, and retinal axon guidance [173–179]. In addition, the cell-specific disruption of* Hs2st* in the endothelial and myeloid cells enhanced the infiltration of neutrophils due to an increase in their binding to IL-8 and macrophage inflammatory protein-2 [162]. Mice with the specific disruption of* Hs2st* in the liver accumulate plasma triglycerides and the uptake of very-low-density lipoproteins is reduced, whereas mice with the specific disruption of* Hs6st* in the liver do not. These findings suggest that the clearance of plasma lipoproteins is dependent on the 2-*O*-sulfation of HS [153].

### 4.16. Hs3st1

*HS3ST1* ^−/−^ mice display normal development and anticoagulant activity [184]; however, it was previously demonstrated that the GlcN 3-*O*-sulfate structure was essential for the anticoagulant activity of heparin and HS [252]. Other HS3ST family members such as HS3ST2, HS3ST3a, HS3ST3b, HS3ST4, HS3ST5, and HS3ST6 may compensate for the loss of HS3ST1 [184].

### 4.17. *Hs6st1* and* Hs6st2*

*HS6ST1*-null mice die during the late embryonic stage, are smaller than the wild type at birth, and have defective retinal axon guidance due to the disturbance of Slit-Robo signaling [177, 178, 181]. In contrast,* HS6ST2*-deficient mice develop normally [183]. However, serum levels of thyroid-stimulating hormone and the thyroid hormone, thyroxin, are higher and lower, respectively, in the deficient mice, which cause a reduction in energy metabolism with an increase in body weight [183]. The storage of mast cell proteases is altered in double knockout mice with* HS6ST1* ^−/−^/*HS6ST2* ^−/−^ [182], and their embryonic fibroblasts are partially defective in FGF signaling [253].

### 4.18. *Sulf1* and* Sulf2* (HS 6-*O*-endosulfatase)

*Sulf1* ^−/−^ mice exhibit no apparent abnormalities [185]. On the other hand,* Sulf2* ^−/−^ mice have a smaller body size and mass [185, 192]. Mice deficient in both* Sulf1* and* Sulf2* have multiple defects including skeletal and renal malformations, which result in neonatal lethality [186]. HS 6-*O*-sulfation and/or desulfation by Sulfs are known to be involved in the cartilage homeostasis mediated by bone morphogenetic protein and Fgf [187], dentinogenesis through Wnt signaling [188], neurite outgrowth mediated by glial cell line-derived neurotrophic factor [189], muscle regeneration [190], and brain development [191]. These findings indicate that the fine-tuning of 6-*O*-sulfation by Sulfs may control multiple functions of HS chains during morphogenesis.

## 5. Human Disorders Affecting the Skeleton and Skin due to the Disturbance of GAGs

### 5.1. PAPSS2

Spondyloepimetaphyseal dysplasia of Pakistani type, which is characterized by kyphoscoliosis, generalized brachydactyly, short and bowed lower limbs, and enlarged knee joints, is caused by mutations in* PAPSS2*: p.Ser438X and p.Arg329X [43, 44].

Patients with mutations in* PAPSS2*, resulting in the substitution of corresponding amino acids (p.Thr48Arg, p.Arg329X, and p.Ser475X), also have spondylodysplasia and premature pubarche, which are accompanied by a short stature, bone dysplasia, excess androgens, hyperandrogenic anovulation, and the loss of dehydroepiandrosterone sulfate [45]. Sulfotransferase 2A1 has been shown to transfer a sulfate group from PAPS to dehydroepiandrosterone (DHEA) in the adrenal glands and liver, resulting in the formation of DHEA-sulfate [254]. The inactivation of PAPSS2 inhibits of not only the formation of PAPS but also the conversion of DHEA into DHEA-sulfate, which leads to the accumulation of DHEA in patients [45]. Excess DHEA is finally converted to testosterone through androsterone.

Autosomal recessive brachyolmia, which is a heterogeneous group of skeletal dysplasias and primarily affects the spine, is also caused by* PAPSS2* mutations [46, 47]. Brachyolmia is characterized by a short stature due to a short trunk, irregular endoplates, a narrow intervertebral disc, calcification of cartilage in the ribs, a short femoral neck and metacarpals, and normal intelligence [46–48]. However, the excess amount of androgens cannot be detected in these patients. Furthermore, PAPS synthase activity was absent in the recombinant mutant enzymes, including p.Cys43Tyr, p.Leu76Gln, and p.Val540Asp [47].

### 5.2. XYLT1

Mutation in* XYLT1* causes an autosomal recessive short stature syndrome characterized by alterations in the distribution of fat, intellectual disabilities, and skeletal abnormalities including a short stature and femoral neck, thickened ribs, plump long bones, and distinct facial features [56]. The homozygous mutation in* XYLT1* gives rise to the substitution of the amino acid, p.Arg481Trp in the deduced catalytic domain, which results in decorin without a DS side chain in addition to mature decorin-PG with a DS chain from the fibroblasts of the patient [56]. In addition, the mutant XYLT1 is diffusely localized in the cytoplasm and partially in the Golgi in the fibroblasts of the patient.

Desbuquois dysplasia type 2 is a multiple dislocation group of skeletal disorders that is characterized by a short stature, joint laxity, and advanced carpal ossification [57]. Five distinct* XYLT1* mutations have been identified to date, including a missense substitution (p.Arg598Cys), nonsense mutation (p.Arg147X), truncated form mutation (p.Pro93AlafsX69), and two splice site mutations [58]. Furthermore, fibroblasts from the affected individuals synthesized a smaller amount of CS and/or DS than those from healthy controls [58].

### 5.3. *B4GALT7* (GalT-I)

Ehlers-Danlos syndrome is a heterogeneous group of heritable connective tissue disorders characterized by joint and skin laxity as well as tissue fragility. Six major types (classical, hypermobility, vascular, kyphoscoliosis, arthrochalasia, and dermatosparaxis types) and several minor types, including the progeroid type, are currently known [255]. Mutations in* B4GALT7* encoding GalT-I cause Ehlers-Danlos syndrome-progeroid type 1, which is characterized by an aged appearance, hypermobile joints, loose yet elastic skin, hypotonic muscles, craniofacial dysmorphism, a short stature, developmental delays, generalized osteopenia, and defective wound healing [61–64]. Galactosyltransferase activity is reduced in the mutant enzymes, p.Arg270Cys, p.Ala186Asp, p.Leu206Pro, and p.Arg270Cys, which results in the lack of DS side chains on decorin and biglycan core proteins and also smaller CS and HS side chains on other PGs [64–68].

A homozygous mutation in* B4GALT7* (p.Arg270Cys) causes a variant of Larsen syndrome in Reunion Island in the southern Indian Ocean, which is called Larsen of Reunion Island syndrome, and is characterized by distinctive facial features, multiple dislocations, dwarfism, and hyperlaxity [69].

### 5.4. B3GALT6 (GalT-II)

Ehlers-Danlos syndrome-progeroid type 2 is caused by mutations in* B3GALT6* encoding GalT-II [70, 71]. GalT-II activity by the mutant enzyme (p.Ser309Thr) is significantly decreased, leading to the loss of GAG chains on the core proteins of various PGs [70]. The autosomal-recessive disorder, spondyloepimetaphyseal dysplasia with joint laxity type 1, which is characterized by hip dislocation, elbow contracture, clubfeet, platyspondyly, hypoplastic ilia, kyphoscoliosis, metaphyseal flaring, and craniofacial dysmorphisms such as prominent eyes, blue sclera, a long upper lip, and small mandible with cleft palate, is also caused by mutations in* B3GALT6* [70–72, 256]. Skeletal and connective abnormalities in both Ehlers-Danlos syndrome-progeroid type 2 and spondyloepimetaphyseal dysplasia with joint laxity type 1 overlap; however, these individuals have no common mutations among fifteen different mutations [70]. The GalT-II activities of the recombinant enzymes, p.Ser65Gly-, p.Pro67Leu-, p.Asp156Asn-, p.Arg232Cys-, and p.Cys300Ser-B3GALT6, were shown to be significantly lower than those of wild-type-B3GALT6 [70]. The mutation that affected the initiation codon, c.1A>G (p.Met1?), for* B3GALT6* resulted in a lower molecular weight of the recombinant protein than that of the wild-type protein with the deletion of 41 amino acids at the N-terminus, which indicated a shift in translation at the initiation codon at the second ATG [70]. Although wild-type B3GALT6 is expressed in the Golgi, the mutant enzyme (p.Met1?) is localized in the nucleus and cytoplasm [70], indicating that the mutant protein may not be functional due to its cellular mislocalization.

### 5.5. *B3GAT3* (GlcAT-I)

A mutation (p.Arg277Gln) in the* B3GAT3* gene encoding GlcAT-I is known to cause Larsen-like syndrome [73, 74], which is characterized by dislocations in the hip, knee, and elbow joints, equinovarus foot deformities, and craniofacial dysmorphisms such as a flattened midface, depressed nasal bridge, hypertelorism, and a prominent forehead [257, 258]. These patients mainly have elbow dislocations with congenital heart defects including a bicuspid aortic valve in addition to characteristic symptoms of Larsen-like syndrome [73]. The p.Arg277Gln mutation results in a marked reduction in GlcAT-I activity in the fibroblasts of these patients and the recombinant enzyme protein [73]. Mature decorin-PG, which is secreted by fibroblasts and has a single DS side chain, was observed in the fibroblasts of healthy controls [73]. On the other hand, fibroblasts from patients generate both a PG form of decorin and DS-free decorin [73]. Moreover, the number of CS and HS in the patients' cells is also reduced.

### 5.6. CSGALNACT1

Neuropathies including Guillan-Barré syndrome, chronic inflammatory demyelinating polyneuropathy, hereditary motor sensory neuropathy, and unknown etiologies are partially caused by mutations in* CSGALNACT1* encoding GalNAcT-I and GalNAcT-II [83]. The GalNAcT-II activities of the recombinant enzymes, CSGalNAcT1-His234Arg and -Met509Arg, were shown to be markedly reduced [83], which indicated that affect in CS chains on PGs in the nervous system may lead to peripheral neuropathies.

### 5.7. CHSY1

Patients with mutations in* CHSY1* have Temtamy preaxial brachydactyly syndrome, which is an autosomal recessive congenital syndrome characterized by facial dysmorphism, dental anomalies, brachydactyly, hyperphalangism, growth retardation, deafness, and delayed motor and mental developments [77, 78]. Their mutations result in the substitution of amino acids and truncation of the CHSY1 protein including p.Gly5AlafsX30, p.Gly19-Leu28del, p.Glu33SerfsX1, p.Gln69X, and p.Pro539Arg and a splice-site mutation [77–79]. A heterozygous mutation in* CHSY1* (p.Phy362Ser) was recently identified in a patient with neuropathy [80].

### 5.8. CHST3 (C6ST1)

Spondyloepiphyseal dysplasia Omani type, which is characterized by severe chondrodysplasia with major involvement of the spine, is caused by a loss-of-function mutation in* C6ST1* [108–113]. Patients with the substitution of amino acid in C6ST1, p.Arg304Gln, have severe kyphoscoliosis, a short stature, mild brachydactyly, rhizomelia, fusion of the carpal bones, and osteoarthritis in the elbow, wrist, and knee joints [108, 109]. Furthermore, additional clinical features, including deafness, metacarpal shortening, and aortic regurgitations due to ventricular septal, mitral, and/or tricuspid defects, have been reported in Turkish siblings who had different mutations in* C6ST1 *(p.Tyr141Met and p.Leu286Pro) [110, 111]. Mutant enzymes of the recombinant C6ST1 and enzymes from the patients' fibroblasts had markedly reduced C6ST activity, which resulted in the loss of chondroitin 6-*O*-sulfate in the fibroblasts [109–111]. Moreover, chondrodysplasia with multiple dislocations, Desbuquois syndrome, autosomal recessive Larsen syndrome, and humero-spinal dysostosis have been attributed to distinct* CHST3* mutations (p.Leu259Pro, p.Arg222Trp, p.Leu307Pro, p.Tyr201X, p.F206X, p.Glu372Lys, p.Gly363AlafsX30, and a mutation at the splice site) [112, 113]. Different pathological diagnoses may be caused by the relatively narrow clinical features and age-related descriptions of the same conditions [113].

### 5.9. *CHST14* (D4ST1)

Ehlers-Danlos syndrome musculocontractural type 1, which is characterized by progressive joint and skin laxity, multiple congenital contractures, progressive multi-system complications, and characteristic craniofacial features, is caused by mutations in* CHST14* encoding D4ST1 (p.Val49X, p.Lys69X, p.Pro281Leu, p.Cys289Ser, p.Tyr293Cys, and p.Glu334GlyfsX107) [97–102]. A recent study described a case of Ehlers-Danlos syndrome musculocontractural type 1 (p.Val49X) in which muscle hypoplasia and weakness was observed, which resulted in myopathy based on laboratory findings such as muscle biopsy, ultrasound, and electromyography [103].

The recombinant mutants of D4ST1 (p.Pro281Leu, p.Cys289Ser, and p.Tyr293Cys) and fibroblasts from affected individuals have markedly reduced sulfotransferase activity [99]. Furthermore, a single DS side chain on decorin-PG from the fibroblasts of patients was found to be replaced by a CS chain, but not dermatan [99]. Immature decorin-PG results in the dispersion of collagen bundles in the dermal tissues of patients.

The autosomal recessive disorder, adducted thumb-clubfoot syndrome, which is characterized by an adducted thumb, clubfoot, craniofacial dysmorphism, arachnodactyly cryptorchidism, an atrial septal defect, kidney defects, cranial ventricular enlargement, and psychomotor retardation, is also caused by mutations in* CHST14* (p.Val49X, p.Arg135Gly, p.Leu137Gln, p.Arg213Pro, and p.Tyr293Cys) [104–106]. The fibroblasts of these patients lack DS chains and have an excess amount of CS chains.

### 5.10. DSE

A mutation in* DSE* (p.Ser268Leu) has been shown to cause Ehlers-Danlos syndrome musculocontractural type 2 [87]. Clinical features including hypermobility of the finger, elbow, and knee joints, characteristic facial features, contracture of the thumbs and feet, and myopathy have been observed in these patients. Epimerase activity is markedly reduced not only in the recombinant mutant DSE (p.Ser268Leu) but also in the cell lysate from these patients [87]. In addition, a decrease in the biosynthesis of DS accompanied by an increase in that of CS has been reported in the fibroblasts of these patients. The deficiencies associated with DSE in addition to D4ST1 affect the biosynthesis of DS, which implies that both enzymes are essential for the development of skin and bone as well as the maintenance of their extracellular matrices.

## 6. Conclusions

The biological roles of CS, DS, and HS chains* in vivo* have been revealed by examining knockout mice in addition to nematodes, fruit flies, and zebrafish [4, 8, 12–14]. However, the mice deficient in glycosyltransferases or sulfotransferases involved in the biosynthesis of GAGs showed embryonic lethality or death shortly after the birth. These observations indicate that GAGs or PGs are essential for early development. Furthermore, studies using the conditional knockout mice have revealed the specific functions of GAGs in individual organs. Recent advances in the study of human genetic diseases in the bone and connective tissue have also clarified the biological significance of the GAG side chains of PGs [7, 14, 20]. The clinical manifestations in human disorders caused by deficiency in the biosynthetic enzymes of GAGs do not always agree with the phenotypes of the deficiency in the corresponding enzymes in knockout mice. This contradiction may be due to the residual enzymatic activity or GAGs in human patients. Although null mutant mice show severe phenotypes including embryonic lethality, human patients appear to show various symptoms depending on the degree of remaining activity of the enzymes. Further comprehensive approaches to the study of molecular pathogeneses involving CS, DS, and HS chains are required to facilitate the development of therapeutics and design of new drugs for these diseases.

## Figures and Tables

**Table 1:** Transporters for UDP-sugars and sulfate as well as biosynthetic enzymes for PAPS.

Transporters and enzymes	Coding genes (synonym)	Chromosomal location	mRNA accession number	MIM number	Human genetic disorders	Clinical features	References for the human diseases	References for the knockout mice
Solute carrier family 26 member A2 (diastrophic dysplasia sulfate transporter)	*SLC26A2 (DTDST) *	5q31–q34	NM_000112	600972 256050 222600 226900	Achondrogenesis type IB Atelosteogenesis type II Diastrophic dysplasia Multiple epiphyseal dysplasia autosomal recessive type	Lethal chondrodysplasia with severe under-development of skeleton, extreme micromelia, death before or immediately after birth. Epiphyseal dysplasia and early onset osteoarthritis.	[38–40]	[41]

Solute carrier family 35 member D1 (UDP-GlcUA/UDP-GalNAc dual transporter)	*SLC35D1 (UGTrel7) *	1p32-p31	NM_015139	269250	Schneckenbecken dysplasia	Neonatal lethal chondrodysplasia, platyspondyly with oval-shaped vertebral bodies, extremely short long bones with dumbbell-like appearance, and small ilia with snail-like appearance.	[42]	[42]

PAPS synthase 2	*PAPSS2 *	10q24	NM_004670 NM_001015880	612847	Spondyloepimetaphyseal dysplasia Pakistani type (PAPSS2 type) Hyperandrogenism Brachyolmia autosomal recessive type	Short, bowed lower limbs, enlarged knee joint, kyphoscoliosis, and mild generalized brachydactyly. Androgen excess, premature pubarche, hyperandrogenic anovulation, low level of serum, dehydroepiandrosterone, short trunk, kyphosis, and scoliosis.	[43–48]	[22, 23, 49–53]

3′-Phosphoadenosine 5′-phosphate 3′-phosphatase	*IMPAD1 (PAPP) *	8q12.1	NM_017813	614078 614010	Chondrodysplasia with joint dislocations GPAPP type	Short stature, chondrodysplasia, with brachydactyly, congenital joint dislocations, cleft palate, and facial dysmorphism.	[54]	[55]

MIM: mendelian inheritance in man.

Among the several transporters and biosynthetic enzymes involved in PAPS and UDP-sugars, some of the mutations that occur have been shown to cause human genetic disorders and are listed here.

**Table 2:** Biosynthetic enzymes of the GAG-linkage region tetrasaccharide.

Enzymes (activity)	Coding genes (synonym)	Chromosomal location	mRNA accession number	MIM number	Human genetic disorders	Clinical features	References for the human diseases	References for the knockout mice
Xylosyltransferase (XylT)	*XYLT1 *	16p12.3	NM_022166	608124	Desbuquois dysplasia type 2, Short stature syndrome	Short stature, joint laxity, advanced carpal ossification, and hand anomalies.	[56–58]	[59]
	*XYLT2 *	17q21.33	NM_022167	608125	—	—	—	[60]

*β*4-Galactosyltransferase-I (GalT-I)	*B4GALT7 *	5q35.2-q35.3	NM_007255	130070 604327	Ehlers-Danlos syndrome progeroid type 1 Larsen of Reunion Island syndrome	Developmental delay, aged appearance, short stature, craniofacial dysmorphism, and generalized osteopenia. Multiple dislocations, hyperlaxity, dwarfism, and distinctive facial features.	[61–69]	—

*β*3-Galactosyltransferase-II (GalT-II)	*B3GALT6 *	1p36.33	NM_080605	271640 615349 615291	Ehlers-Danlos syndrome progeroid type 2 Spondyloepimetaphyseal dysplasia with joint laxity type 1	Sparse hair, wrinkled skin, defective wound healing with atrophic scars, osteopenia, and radial head dislocation. Spatulate finger with short nail, hip dislocation, elbow contracture, clubfeet, and mild craniofacial dysmorphism including prominent eye, blue sclera, long upper lip, and small mandible with cleft palate.	[70–72]	—

*β*3-Glucuronyltransferase-I (GlcAT-I)	*B3GAT3 *	11q12.3	NM_012200	245600 606374	Larsen-like syndrome B3GAT3 type Multiple joint dislocations, short stature, craniofacial dysmorphism, and congenital heart defects	Joint dislocations mainly affecting the elbow, congenital heart defects such as bicuspid aortic valve, aortic root dilatation.	[73, 74]	[75, 76]

Xylose 2-*O*-kinase	*FAM20B* (*GXK1*)	1q25	NM_014864	611063	—	—	—	—

Xylose 2-*O*-phosphatase	*ACPL2* *(XYLP) *	3q23	NM_152282	—	—	—	—	—

—, not reported.

B4GALT7: xylosylprotein beta 1,4-galactosyltransferase 7; B3GALT6, beta 1,3-galactosyltransferase 6; B3GAT3, beta 1,3-glucuronyltransferase 3; FAM20B, Family with sequence similarity 20 member B; ACPL2, acid phosphatase-like 2.

**Table 3:** Biosynthetic enzymes of CS and DS chains.

Enzymes (activity)	Coding genes (synonym)	Chromosomal location	mRNA accession number	MIM number	Human genetic disorders	Clinical features	References for the human diseases	References for the knockout mice
Chondroitin synthase (GalNAcT-II, CS-GlcAT-II)	*CHSY1 *	15q26.3	NM_014918	605282 608183	Temtamy preaxial brachydactyly syndrome Syndromic recessive preaxial brachydactyly Neuropathy	Short stature, limb malformation, hearing loss.	[77–80]	[81]
	*CHSY2* (*CHSY3, CSS3*)	5q23.3	NM_175856	609963	—	—	—	—
	*CHSY3* (*CHPF2, CSGLCA-T*)	7q36.1	NM_019015	608037	—	—	—	—

Chondroitin-polymerizing factor (GalNAcT-II, CS-GlcAT-II)	*CHPF* (*CSS2*)	2q35	NM_024536	610405	—	—	—	[81, 82]

Chondroitin *N*-acetylgalactosaminyltransferase (GalNAcT-I, GalNAcT-II)	*CSGALNACT1 *	8p21.3	NM_018371	—	Hereditary motor and sensory neuropathy Unknown type Bell's palsy	Intermittent postural tremor, reduction in compound muscle action potentials, acquired idiopathic generalized anhidrosis, hemifacial palsy.	[83]	[81, 84–86]
	*CSGALNACT2 *	10q11.21	NM_018590	—	—	—	—	[86]

Dermatan sulfate epimerase	*DSE *	6q22	NM_013352	615539 605942	Ehlers-Danlos syndrome musculocontractural type 2	Characteristic facial features, congenital contractures of the thumbs and the feet, hypermobility of finger, elbow, and knee joints, atrophic scarring of the skin, and myopathy.	[87]	[88, 89]
	*DSEL *	18q22.1	NM_032160	611125	Bipolar disorder Depressive disorder Diaphragmatic hernia Microphthalmia	Alternating episodes of depression and mania or hypomania, and congenital malformation of the diaphragm.	[90–92]	[93]

Uranyl 2-*O*-sulfotransferase	*UST *	6q25.1	NM_005715	610752	—	—	—	—

Chondroitin 4-*O*-sulfotransferase	*CHST11* (*C4ST-1*)	12q	NM_018413	610128	—	—	—	[94–96]
	*CHST12* (*C4ST-2*)	7p22	NM_018641	610129	—	—	—	—
	*CHST13* (*C4ST-3*)	3q21.3	NM_152889	610124	—	—	—	—

Dermatan 4-*O*-sulfotransferase	*CHST14* (*D4ST-1*)	15q15.1	NM_130468	601776 608429	Ehlers-Danlos syndrome musculocontractural type 1 Adducted thumb-clubfoot syndrome	Craniofacial dysmorphism, multiple contractures, progressive joint and skin laxities, multisystem fragility-related manifestations, contractures of thumbs and feet, defects of heart, kidney and intestine.	[97–106]	[96, 107]

Chondroitin 6-*O*-sulfotransferase	*CHST3* (*C6ST-1*)	10q22.1	NM_004273	143095 603799	Spondyloepiphyseal dysplasia with congenital joint dislocations Spondyloepiphyseal dysplasia Omani type Chondrodysplasia with multiple dislocations Humerospinal dysostosis Larsen syndrome autosomal recessive type Desbuquois syndrome	Short stature, severe kyphoscoliosis, osteoarthritis (elbow, wrist and knee), secondary dislocation of large joints, rhizomelia, fusion of carpal bones, mild brachydactyly, metacarpal shortening, ventricular septal defect, mitral and tricuspid defects, aortic regurgitations, deafness.	[108–113]	[114–116]

*N*-Acetylgalactosamine-4-sulfate- 6-*O*-sulfotransferase	*CHST15* (*GalNAc4S-6ST*)	10q26	NM_015892	608277	—	—	—	[117]

—: not reported.

CSS: chondroitin sulfate synthase; DSEL: dermatan sulfate epimerase-like; CHST: carbohydrate sulfotransferase.

**Table 4:** Biosynthetic enzymes of HS and heparin chains.

Enzymes (activity)	Coding genes (synonym)	Chromosomal location	mRNA accession number	MIM number	Human genetic disorders	Clinical features	References for the human diseases	References for the knockout mice
Exostosin (GlcA and GlcNAc transferases)	*EXT1 *	8q24.11	NM_000127	133700 215300 608177	Exostoses multiple type 1 Chondrosarcoma	The formation of cartilage-capped tumors (exostoses) that develop from the growth plate of endochondral bones, especially of long bones.	[118]	[119–135]
	*EXT2 *	11p12-p11	NM_000401	133701 608210	Exostoses multiple type 2	Same as above.	[118]	[136]

Exostosin-like 2 (GlcNAc transferase-I)	*EXTL2 *	1p21	NM_001439	602411	—	—	—	[137, 138]

Exostosin-like 1 (GlcNAc transferase-II)	*EXTL1 *	1p36.1	NM_004455	601738	—	—	—	—

Exostosin-like 3 (GlcNAc transferase-I and -II)	*EXTL3 *	8p21	NM_001440	605744	—	—	—	[139]

GlcNAc *N*-deacetylase and *N*-sulfotransferase	*NDST1 *	5q33.1	NM_001543	600858	—	—	—	[140–164]
	*NDST2 *	10q22	NM_003635	603268	—	—	—	[165–167]
	*NDST3 *	4q26	NM_004784	603950	—	—	—	[168]
	*NDST4 *	4q26	NM_022569	615039	—	—	—	—

HS GlcUA C5-epimerase	*GLCE *	15q23	NM_015554	612134	—	—	—	[169–172]

HS 2-*O*-sulfotransferase	*HS2ST1 *	1p22.3	NM_012262	604844	—	—	—	[153, 162, 173–179]

HS 6-*O*-sulfotransferase	*HS6ST1 *	2q21	NM_004807	614880 604846	Hypogonadotropic hypogonadism 15 with or without anosmia	Lack of sexual maturation and low levels of circulating gonadotropins and testosterone.	[180]	[177, 178, 181, 182]
	*HS6ST2 *	Xq26.2	NM_147174 NM_147175	300545	—	—	—	[182, 183]
	*HS6ST-3 *	13q32.1	NM_153456	609401	—	—	—	—

HS 3-*O*-sulfotransferase	*HS3ST1 *	4p16	NM_005114	603244	—	—	—	[184]
	*HS3ST2 *	16p12	NM_006043	604056	—	—	—	—
	*HS3ST3A1 HS3ST3B1 *	17p12	NM_006042 NM_006041	604057 604058	—	—	—	—
	*HS3ST4 *	16p11.2	NM_006040	604059	—	—	—	—
	*HS3ST5 *	6q22.31	NM_153612	609047	—	—	—	—
	*HS3ST6 *	16p13.3	NM_001009606	—	—	—	—	—

HS 6-*O*-endosulfatase	*SULF1 *	8q13.2-q13.3	NM_015170	610012	—	—	—	[185–191]
	*SULF2 *	20q12–q13.2	NM_018837 NM_198596	610013	—	—	—	[185–192]

—: not reported.

## Abbreviations

B3GALT6: Beta 1,3-galactosyltransferase 6
*B4GALT7*: Beta 1,4-galactosyltransferase 7
*B3GAT3*: Beta-1,3-glucuronyltransferase 3
C4ST: Chondroitin 4-*O*-sulfotransferase
C6ST: Chondroitin 6-*O*-sulfotransferase
CHST: Carbohydrate sulfotransferase
CHSY: Chondroitin synthase
CS: Chondroitin sulfate
CSGALNACT: Chondroitin sulfate* N*-acetylgalactosaminyltransferase
D4ST: Dermatan 4-*O*-sulfotransferase
DS: Dermatan sulfate
DSE: Dermatan sulfate-glucuronyl C5-epimerase
DSEL: Dermatan sulfate epimerase-like
GAG: Glycosaminoglycan
GalNAc: * N*-Acetyl-d-galactosamine
GalNAc4S-6ST: * N*-Acetyl-d-galactosamine 4-sulfate 6-*O*-sulfotransferase
GlcUA: d-Glucuronic acid
HS: Heparan sulfate
IdoUA: l-Iduronic acid
PAPS: 3′-Phosphoadenosine 5′-phosphosulfate
PAPSS2: 3′-Phosphoadenosine 5′-phosphosulfate synthase 2
PG: Proteoglycan.
